# Liposome-based drug delivery systems for skin wound healing: a promising drug delivery strategy

**DOI:** 10.3389/fbioe.2026.1756872

**Published:** 2026-02-12

**Authors:** Zhensheng Ma, Kaiying Zhang, Jiayu Luo, Shuoling Chen, Shenglong Tan, Dandan Ma

**Affiliations:** Department of Endodontics, Stomatological Hospital, School of Stomatology, Southern Medical University, Guangzhou, China

**Keywords:** Biomedical and pharmaceutical applications, drug delivery, liposome biomaterials, skin wound healing, topical administration

## Abstract

Skin wound healing remains a significant challenge in clinical medicine. Liposomes (LPs), as a versatile drug delivery system, have garnered widespread attention for their potential in promoting skin wound healing. However, the limitations of conventional LPs have hindered their broader clinical applications. To enhance the efficacy of LPs, researchers have developed various liposome-based delivery systems (LPs-DS) by integrating different materials and technologies. This review focuses on the field of skin wounds, highlighting the advantages of LPs-DS and clinical translational concepts in promoting skin wound healing. It summarizes their applications in different types of wounds and suggests potential future applications, aiming to provide a reference for further research on drug delivery systems.

## 1 Introduction

The skin serves as the first line of defense against external damage (Gravitz, 2018). When the integrity of the skin is compromised, its barrier function is lost, potentially leading to deep tissue damage or internal microenvironmental disorders (Luo et al., 2023). Skin wounds can result from cuts, burns, and various skin diseases (Yu et al., 2014). Globally, skin wounds are one of the most prevalent public health concerns, with market expenditures exceeding $20.59 billion. Despite this, over one billion people continue to suffer from wounds (Motsoene et al., 2023), and these numbers are expected to grow as the population ages and the prevalence of systemic diseases, such as diabetes, increases (Meng et al., 2018). Research into the treatment of skin wounds is thus both clinically crucial and cost-effective.

Currently, the main strategies for managing skin wounds include debridement, skin grafting, and topical therapeutic medications (Wang et al., 2019). However, severe burns, infected wounds, and other hard-to-heal skin wounds can impair the physiological function of the skin and may even lead to death. Despite extensive research aimed at improving skin wound care, the clinical management of chronic wounds remain unsatisfactory (Kim et al., 2019). Successful wound healing depends not only on the therapeutic agents applied but also on the wound healing materials that protect the wound surface and regulate the local healing environment (Boateng et al., 2008). As a result, contemporary wound care strategies increasingly focus on combining bioactive therapies with functional wound dressings rather than administering free drugs alone (Guo and Dipietro, 2010). A wide range of materials has been developed, including hydrogels, nanofibrous membranes, films, sponges, foams, and three-dimensional scaffolds, which provide both structural support and a permissive microenvironment (Lee and Mooney, 2012). In this context, drug delivery and localization is particularly beneficial, especially for chronic wounds. Liposome has therefore attracted considerable attention as versatile carriers for a wide range of therapeutic agents and are most often incorporated into wound healing materials to enhance local delivery efficacy (Akbarzadeh et al., 2013). Within such integrated systems, liposome and wound healing materials serve complementary but distinct functions. Liposome primarily govern drug encapsulation, release behavior, and interactions with target cells, while the surrounding materials provide mechanical stability, spatial confinement, and a biologically favorable interface with the wound bed (Bozzuto and Molinari, 2015). Accordingly, an understanding of liposome is essential.

In recent years, drug delivery systems have shown great potential in accelerating wound healing (Kim et al., 2019). These systems are advanced dosage forms designed for the prevention and treatment of diseases (Cai et al., 2021), emphasizing the appropriate timing and dosage of administration (Zhou et al., 2022). Among these delivery vehicles, liposomes (LPs) are closed vesicles with monolayer or bilayer structures formed by lecithin or other lipids, used for delivering various biomolecules (Cheng et al., 2020). Traditional LPs, also known as first-generation LPs (Cao et al., 2022), offer several advantages, including excellent biocompatibility, biodegradability, enhanced drug bioavailability, and decreased side effects (Peng et al., 2022). Continuous innovation has led to the testing of many products in various clinical trials, including anticancer agents (Dymek and Sikora, 2022), antifungal agents (Forssen, 1997). Following small molecule drugs and antibodies, the integration of the third-generation drug (nucleic acid drugs) with LPs has experienced rapid advancement (Weng et al., 2020). Nonetheless, traditional LPs present challenges in clinical application, such as limited drug stability, short blood half-life, restricted clearance capacity, inadequate physical characteristics, complex production process and insufficient targeting (Clarke et al., 2006). Consequently, researchers in this field are engaging in more promising studies to address these shortcomings.

For enhanced efficacy of LPs, researchers have integrated various materials and technologies to adjust biological properties, creating systems with specific properties unattainable by a single component. In this review, liposome-based delivery systems (LPs-DS) refer to complexes formed by combining basic LPs with different materials and technologies. The versatility of liposome assembly allows for the preparation of different phospholipids with distinct functions. Utilizing pH, temperature responsive, or light responsive phospholipids for liposome construction enables tailored environmental response reactions. Moreover, surface conjugation of LPs with various elements enhances targeted applications, departing from the outer surface of traditional LPs (Wang et al., 2024). Notably, scholars have reviewed the progress of polymer-modified LPs in drug delivery, emphasizing changes in properties and factors impacting therapeutic outcomes (Cao et al., 2022). Strategies to enhance liposome-mediated targeting, uptake, and therapeutic response through surface modifications have been explored (Le et al., 2019). Collaborating with different substances enables LPs-DS to use their individual strengths and play complementary roles. Gradually integrated into pharmaceutical research, LPs-DS find applications in intestinal targeting (Zhang et al., 2023), osteoarthritis (Bordon et al., 2023), neurodegenerative diseases (Passeri et al., 2022), retinal diseases (Urquhart and Eriksen, 2019), particularly in tumor treatments like breast cancer (Wang et al., 2020), pancreatic cancer (Raza et al., 2023), glioma (Park, 2018). Additionally, surface-modified LPs have been investigated in cancer diagnosis research (Llop and Lammers, 2021). Scholars emphasize the importance of an ideal liposome delivery system for oncology akin to a multi-stage rocket, combining multiple functions to target the tumor environment specifically, bind to tumor cells, and act as a drug release agent (He and Tang, 2018). Recognizing the limitations of conventional LPs with singular properties, clinical trials are swiftly shifting focus towards functional LPs-DS (Aronson et al., 2021). In the field of skin diseases, studies have highlighted the role of LPs-DS in treating skin leishmaniasis (Ortega et al., 2017), skin inflammation (How et al., 2020). However, there is currently no summary of the progress in this field, this review focuses on skin wound healing, delineating various types of skin wounds, their healing processes, current healing promotion measures, advantages of LPs-DS and clinical translational concepts in skin wound healing promotion, and applications in different types of skin wounds, aiming to offer insights for future clinical targeting treatments. Finally, it addresses future challenges and perspectives in this field.

## 2 Introduction of skin wound

### 2.1 Classification and healing process of skin wound

Skin wounds are generally classified as acute wounds, which typically undergo complete functional tissue repair within 3 weeks and are often caused by trauma or surgery (Alghamdi, 2023); and chronic wounds, lasting at least 3 months and prone to complications (Gould et al., 2015). Based on their etiology, skin wounds can be categorized into traumatic wounds (such as incisions or excision wounds), thermal injuries (including burn or frostbite wound), infected wound, metabolic-associated chronic wounds (diabetic wound) and vascular wound (venous or arterial). Each category is characterized by distinct pathological mechanisms and healing challenges. The wound microenvironment varies depending on the cause, with treating skin wounds in diabetic patients presenting greater challenges due to elevated levels of reactive oxygen species and inflammation (Kasiewicz and Whitehead, 2016). This classification holds significant clinical value for precision.

The process of skin wound healing typically involves four interconnected phases: hemostasis, inflammation, proliferation, and remodeling (Sun et al., 2014). After a skin injury, platelets and blood clots initially control excessive bleeding and create an early extracellular matrix for cell attachment and proliferation. Concurrently, the release of chemokines by blood clots and damaged cells attracts inflammatory cells from surrounding tissues and the bloodstream to the injury site (Mazurek et al., 2022). As the inflammatory response diminishes, dermal and epidermal cells migrate and proliferate, marking the start of the proliferative phase. During this phase, endothelial cells become activated, the endothelial basement membrane degrades, vascularization of wound margins occurs, and fibroblasts migrate to the wound site in response to factors like PDGF, TGF-β1, and FGF. Fibroblasts proliferate and secrete abundant extracellular matrix, which partially transforms into myofibroblasts responsible for wound contraction and matrix deposition, leading to the formation of granulation tissue comprising capillaries, fibroblasts, and extracellular matrix (Kim et al., 2019). Keratinocytes also migrate to the wound bed, facilitating wound coverage with new epidermis to achieve re-epithelialization (Zhu et al., 2020). Finally, the remodeling phase involves a transition in the dermis from predominance of type III to type I collagen until a healthy tissue balance is attained (Sun et al., 2014).

**FIGURE 1:**
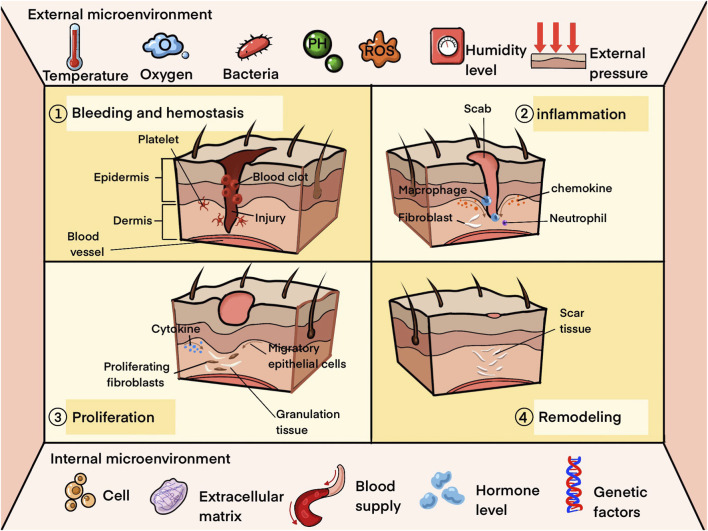
Healing process and microenvironment of skin wounds.

Chronic wounds typically exhibit disordered and prolonged healing processes, necessitating persistent chronic inflammation that exacerbates tissue damage (Chin et al., 2019). Both local and systemic factors play crucial roles in wound healing (Mazurek et al., 2022), with various studies demonstrating factors that disrupt the progression of wound healing, such as cytokine dysregulation, heightened oxidative stress, and the formation of bacterial biofilms (Chin et al., 2019; Darwin and Tomic-Canic, 2018; Jari and Praseetha, 2022). Moreover, the microenvironmental theory of wound healing emphasizes the dynamic interplay between the external microenvironment (e.g., temperature, pH, microorganisms) directly surrounding the wound and the internal microenvironment (e.g., cells, extracellular matrix) beneath the wound surface, influencing the overall healing process (Figure 1) (Li et al., 2022). It is evident that wounds of diverse origins exhibit distinct influences, underscoring the therapeutic importance of classifying wounds based on their specific etiologies.

### 2.2 Conventional strategies for the treatment of skin wound

**FIGURE 2:**
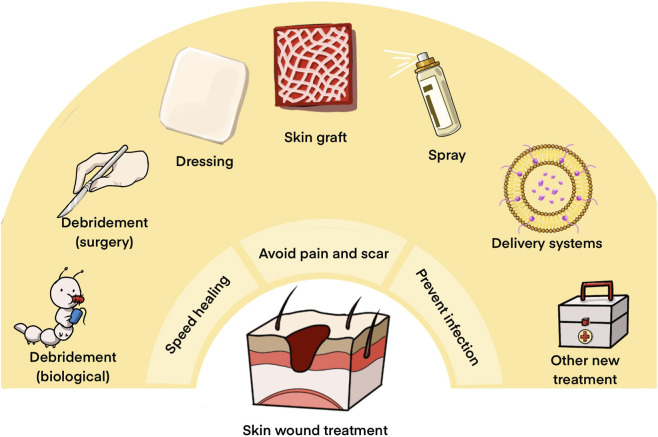
The ultimate goal of wound management and strategies for the treatment.

Traditional wound management strategies aim to prevent infection, expedite healing, and minimize pain and scarring (Wang et al., 2019). Presently, key approaches for skin wound care encompass debridement, skin grafting, and topical medications (Darwin and Tomic-Canic, 2018) (Figure 2). Emerging therapies are increasingly significant in managing complex wounds (Ding J. Y. et al., 2023).

#### 2.2.1 Debridement

Debridement serves as the quickest means of eliminating non-viable tissue from the wound bed, transitioning chronic wounds to acute wounds. It effectively removes necrotic tissue, reducing infection risks. However, debridement has drawbacks, including potential pain and secondary trauma (Bradley et al., 1999; Kammerlander et al., 2005).

#### 2.2.2 Skin grafts

Skin grafting involves transferring skin tissue from one area to another (Sun et al., 2014) and comprises living and bioengineered skin grafts. Autologous and allogeneic grafts are commonly utilized, with autologous grafts posing no risk of rejection but necessitating stringent criteria for use. Allografts face immune rejection issues but are viable as temporary substitutes (Ahmadi-Aghkand et al., 2016; Sun et al., 2014). Ideal bioengineered skin substitutes should prioritize safety, efficacy, and simplicity (Morimoto et al., 2005; Oh et al., 2013), emerging as promising alternatives for skin grafting (Guo and Dipietro, 2010).

#### 2.2.3 Topical medications

Topical medications, such as wound dressings and sprays, are prevalent treatments for promoting healing and preventing infection (Wang et al., 2018; Wang et al., 2019). However, conventional medications often suffer from rapid drug loss, limited penetration into the wound bed, and insufficient local retention (Chin et al., 2019). In this context, liposomes have emerged as particularly attractive carriers for topical wound therapy. Owing to their phospholipid bilayer structure, liposomes exhibit excellent biocompatibility and structural similarity to cellular membranes, enabling efficient encapsulation of both hydrophilic and hydrophobic therapeutics (Schiffelers and Storm, 2008). Moreover, liposomal encapsulation can protect labile drugs from degradation, enhance local drug retention, and reduce systemic exposure, which is especially advantageous for prolonged wound treatment. Importantly, liposomes can be readily integrated into wound dressings or hydrogels and engineered for sustained or stimulus responsive release, allowing better adaptation to the dynamic wound environment (Zununi et al., 2017). However, limitations in the performance of traditional LPs hinder their application, motivating researchers to explore innovative combinations of LPs with diverse materials and technologies, providing the rationale for the focused discussion of LPs-DS in the following section.

## 3 Advantages and clinical translational concept of LPs-DS

**FIGURE 3:**
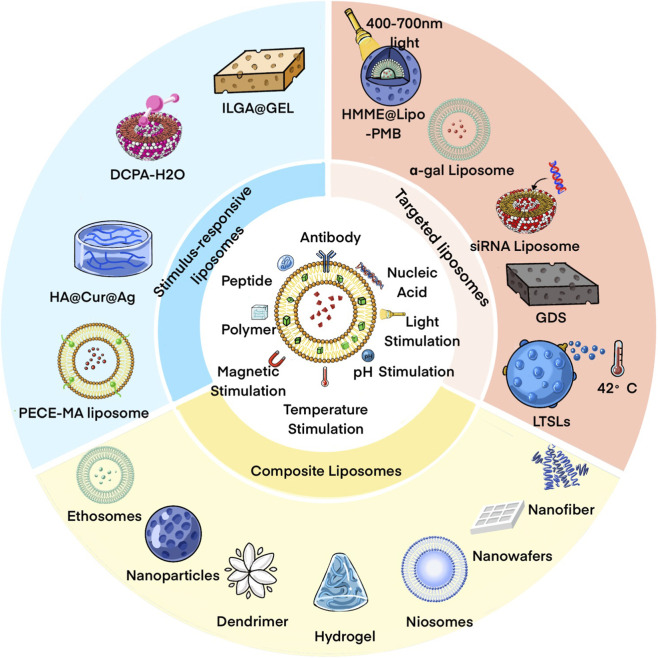
LP-based systems for skin wound healing. The modification of liposome delivery system contains the functional liposomes (stimulus responsive liposomes/targeted liposomes) and liposomes with other outstanding materials (composite liposomes).

LPs are enclosed vesicles with a monolayer or bilayer structure composed of lecithin or other lipids, utilized for transporting a wide array of biomolecules. In an aqueous environment, they create bilayered hollow spheres with hydrophobic tails oriented “tail-to-tail” and hydrophilic heads positioned “shoulder-to-shoulder,” enabling the loading of hydrophilic drugs in the hollow part and lipophilic drugs in the lipid bilayer (Schiffelers and Storm, 2008). It should be noted that the properties are strongly influenced by their preparation and purification techniques (Akbarzadeh et al., 2013). Different preparation methods, such as thin-film hydration, solvent injection, and sonication or extrusion-based techniques, can result in substantial variations in particle size distribution and lamellarity, which in turn affect circulation behavior and tissue penetration (Torchilin, 2005). Similarly, purification strategies including dialysis, centrifugation, and size-exclusion chromatography play a critical role in removing unencapsulated drugs and residual solvents, thereby influencing drug loading efficiency and system stability (Sercombe et al., 2015). In particular, scalable and reproducible manufacturing approaches are increasingly recognized as essential for the successful translation from laboratory studies to clinical use (Allen and Cullis, 2013). Therefore, it is important to consider in preparation and purification strategies rather than from formulation design alone. Serving as drug delivery vehicles, LPs offer numerous advantages, including excellent biocompatibility, biodegradability, enhanced drug bioavailability, and decreased side effects (Zununi et al., 2017). However, they are limited by factors such as overall drug stability and inadequate physical characteristics of the carrier. To enhance the therapeutic effectiveness of LPs, researchers have developed LPs-DS by integrating LPs with suitable materials and technologies, aiming to optimize various biological properties and create systems with specific attributes (Figure 3).

The advantage of LPs-DS lies in their capacity to harness diverse materials and technologies to achieve multifunctionality. Scholars have highlighted that the optimal liposome delivery system should resemble a multistage rocket, integrating various functions (He and Tang, 2018), prompting a shift in numerous clinical trials toward functional LPs-DS (Aronson et al., 2021). Various studies have detailed the formulations of LPs-DS tailored for skin wound healing, showcasing distinctive performance benefits. Initially, as a drug delivery system, LPs have been extensively utilized in clinical and commercial settings, and modifications can expedite their clinical adoption. Pertinently, drug storage, controlled release, and delivery mechanisms are pivotal, with LPs-DS demonstrating exceptional performance in these areas: LPs with a Silk Fibroin Hydrogel Core (Xu et al., 2017) exhibit dual slow-release properties for bFGF, with prolonged activity compared to conventional LPs. On the other hand, hyaluronic acid-modified LPs (Yerushalmi et al., 1994) act as slow-release reservoirs for growth factors, offering controlled release rates and high cell binding affinity, mimicking *in vivo* targets. Moreover, the use of penetration enhancers has been extensively researched to enhance drug penetration and delivery; for instance, 20%–50% ethanol addition enhances drug permeation (Magdy et al., 2022), while nanoLPs containing propylene glycol (Pg) (Kianvash et al., 2017) significantly bolster drug concentrations and extended retention time on the skin. To safeguard less stable drugs during administration, LPs-DS can be fortified with chitosan for enhanced stability (Eid et al., 2022; Mengoni et al., 2017). Furthermore, combining hydrogels with LPs presents an effective approach, as the dispersion of LPs in hydrogels enhances stability through hydrogel network support, concurrently shielding LPs from external factors (Wang S. et al., 2021). This amalgamation not only ensures a moist wound environment to preserve liposome integrity but also prevents their rapid elimination (Pandey et al., 2023), showcasing potential as a carrier system.

In addition, several innovative design concepts have been proposed, including film-forming sprays incorporating LPs and chitosan (Umar et al., 2021), which offer enhanced comfort during application, ensure uniform drug distribution, and form adaptable films that conform to wound textures, facilitating excellent controlled release. Similarly, NIR-II reactive nano-sprayable dressings, integrated with temperature responsive hydrogels (Pan et al., 2023), exhibit excellent fluidity and rapid photothermal transformation, resulting in the formation of adhesive gel films that are highly appealing. Furthermore, the utilization of light-triggered liposomal Ca^2+^ release in combination with enzymes presents a novel approach to wound healing, demonstrating the innovative concept of light-triggered protein hydrogel formation (Sihorwala et al., 2023; Zhang et al., 2002). To address oxygenation challenges in the healing process, a tailored left-shifted liposome hemoglobin vesicle has been engineered (Plock et al., 2009), acting as an artificial oxygen carrier to enhance tissue oxygen distribution. Nanomaterial advancements have led to the application of electrostatic spinning technology in liposome research, embedding LPs in ultrathin electrostatic spinning fibers or creating electrostatic spinning nanocomposite membranes with LPs and chitosan. These developments impart outstanding physical properties, slow-release capabilities, and antioxidant properties (Ding J. Y. et al., 2023; Li et al., 2014). Exploring liposome derivatives has also yielded promising results: Niosomes in conjunction with gelma showcase improved biocompatibility, stability, and controlled drug release (Moghtaderi et al., 2023). The incorporation of hyaluronic acid in transfersomes has resulted in an exceptional drug reservoir with enhanced delivery efficacy and stability (Castangia et al., 2021). Recognizing the impact of liposomal surface charge on carrier physicochemical properties and consequent effects on skin wound healing, the design of ionic LPs has specifically targeted enhancements in skin permeability (Mai et al., 2021; Ternullo et al., 2019; Ternullo et al., 2018).

Nano drug delivery systems utilize nanoparticles as carriers, which, by virtue of their unique physicochemical properties, are able to break through biological barriers, significantly increase the bioavailability of drugs, and effectively reduce nonspecific damage to healthy tissues. Especially in the treatment of localized diseases such as skin wounds, nanoliposomes and polymeric carriers show great potential and have become an important research direction in clinical translational medicine (Kianvash et al., 2017). Targeted drug delivery, on the other hand, improves the precision and efficacy of therapies by precisely delivering drugs to lesions through targeted molecules, such as antibodies or ligands (Wardlow et al., 2016; Galili et al., 2010; Cui et al., 2024; Wigglesworth et al., 2011). Intelligent drug delivery systems regulate the release of drugs based on specific physiological environments (e.g., pH, temperature), realizing precise control over the timing and location of drug release to ensure that the drug is released at the right time and site to maximize its therapeutic effect (Pan et al., 2023; Wang D. Y. et al., 2021; Shi et al., 2024; Liu et al., 2020). Gene drug delivery technology shows a broad application prospect in therapy by efficiently delivering gene therapy drugs to target cells or tissues, thereby altering gene expression (Wang et al., 2023; Rabbani et al., 2017). Notably, liposomes served as the gene carriers in this system, while the dermal substitute functioned as a secondary scaffold for localized delivery. All these technological approaches not only demonstrate the clinical translational potential of drug delivery systems, but also drive therapeutic strategies toward greater precision and personalization. As these conceptual technologies continue to mature and make further breakthroughs in clinical applications, they will provide more efficient and precise solutions in the treatment of many intractable diseases.

In conclusion, the research on LPs-DS is expanding vastly, with various formulations offering distinctive performance advantages. LPs-DS exhibit undeniable performance improvements compared to traditional counterparts, enhancing drug storage, slow release, delivery, and system stability significantly, thereby addressing the limitations of traditional LPs like general drug stability and subpar physical properties. Nevertheless, the key focus for future research lies in identifying the optimal formulation and striking a balance between enhancing performance and ensuring biosafety. We propose that selecting specific LPs-DS tailored to different disease microenvironments is a practical strategy. Anticipating a promising future, the development of multifunctional LPs-DS customized for specific microenvironments, building storage, slow-release, and delivery libraries with optimal performance, and integrating them with advanced technologies for targeted therapies to achieve an ideal multistage rocket therapeutic approach is envisioned.

## 4 LP-based drug delivery strategies for different wound types

While studies have predominantly concentrated on designing LPs-DS for the skin wound healing processes, it is essential to recognize that distinct characteristics are inherent to skin wounds originating from diverse etiologies, leading to unique therapeutic requirements (Li et al., 2022). Tailoring therapeutic elements to the specific microenvironment of individual wound types is a promising approach to enhance healing outcomes and guide targeted clinical interventions effectively. Hence, this section delves into diverse skin wounds to delineate their distinctive characteristics and therapeutic needs. This analysis serves as a foundational overview of recent advancements in research on LPs-DS, offering insights for future precision in clinical treatments.

### 4.1 LP-based systems for infected wound treatment

Infected wounds present a common hurdle in skin wound healing due to the vulnerability of the wound bed and underlying tissues to microbial invasion in the absence of a protective skin barrier. To address this challenge, topical antimicrobials have been empirically utilized to thwart wound infections. Topical administration offers the advantage of delivering drugs directly to the infection site, heightening treatment efficacy compared to systemic treatments (Trengove et al., 1996). Strategic topical application not only minimizes side effects, toxicity, and bacterial resistance but also enhances treatment efficiency by ensuring high antimicrobial concentrations at the infection site (Lio and Kaye, 2009). However, improper utilization may lead to the development of drug-resistant bacterial strains, complicating wound management and potentially prolonging chronic wounds (Rodrigues et al., 2019). Antimicrobial peptides are being explored as promising remedies for skin wound infections, with piggyback delivery systems emerging as a viable research avenue to combat their limitations like low bioavailability and rapid extracellular degradation (Raileanu et al., 2023). Additionally, the integration of silver into wound dressings has garnered attention as a promising field of study (Murphy and Evans, 2012). Although silver sulfadiazine is a common choice, its effectiveness in wound healing lacks conclusive evidence (Miller et al., 2012). Recent advancements have focused on incorporating drugs into wound dressings as drug delivery systems (Han and Ceilley, 2017). While this approach shows promise, caution is necessary as some conventional hydrogel and alginate dressings may induce adverse effects (Barnett and Varley, 1987). Given the challenge of antibiotic resistance (Negut et al., 2018), particularly against methicillin-resistant *Staphylococcus aureus* (MRSA), the development of effective topical wound drug delivery systems is critical (Hiramatsu et al., 1997). These systems should aim to regulate peri-wound moisture, establish a shield against wound pathogens, facilitate targeted drug delivery, assure sustained release of antimicrobial agents, and mitigate potential adverse reactions.

#### 4.1.1 Targeted LP

In the field of drug delivery systems, the precision of drug delivery is paramount for treatment success. Targeted delivery seeks to transport drugs directly to diseased tissues, minimizing systemic toxicity, reducing drug dosages, and enhancing treatment effectiveness. While passive targeting relies on disease-specific environmental cues, active targeting involves binding differentially expressed molecules to relevant ligands, resulting in a structure similar to a lock and key mechanism, such as the case with LPs targeting specific tumor lipids (Bahutair et al., 2022). Extensive research has explored the application of targeted LPs, especially for treating infected skin wounds (Sapra and Allen, 2003). Typically, targeted LPs are functionalized with ligands such as antibodies, peptides, sugars, or aptamers that bind to overexpressed receptors or molecular markers on target cells within the wound microenvironment. At the same time, we need to pay more attention to overall targeting effectiveness as a system. Active targeting strategies offer the clearest advantages under systemic administration, where ligand–receptor interactions can enhance accumulation at the wound site while reducing off-target exposure. In addition, cellular-level targeting within the wound bed, such as macrophage, fibroblast, endothelial cell, or bacteria-specific delivery, represents another scenario in which targeted LPs may provide functional benefits beyond spatial localization alone. Thus, we emphasize more on targeting at the cellular and molecular levels.

Importantly, active targeting introduces additional formulation complexity and cost, including ligand synthesis, conjugation, purification, and quality control of ligand density and orientation. These factors may increase batch-to-batch variability and pose challenges for large-scale manufacturing. As a result, the benefits of targeted LPs must be carefully weighed against these trade-offs, and their application may be most justified for potent therapeutics, expensive biologics, or scenarios requiring precise cellular modulation within the wound microenvironment.A representative example comes from studies on α-gal liposomes (Galili et al., 2010). In this system, liposomes bearing multiple α-gal epitopes bind endogenous anti-Gal in the wound fluid, leading to local activation of the complement cascade, generation of chemotactic factors, and rapid recruitment and activation of macrophages, which orchestrate accelerated healing, enhanced angiogenesis, and reduced scar formation. These effects were markedly greater in genetically engineered mice capable of producing anti-Gal compared to controls lacking this antibody, demonstrating that the therapeutic benefit is dependent on specific ligand–antibody recognition rather than passive localization alone. Experts (Tang et al., 2025) reported a site-specific antibacterial microneedle (MN) platform, in which vancomycin was co-delivered with photoactive black phosphorus quantum dots encapsulated in macrophage membrane–coated cationic liposomes. In MRSA-infected wound model, this strategy reduced bacterial burden and inflammation and accelerated wound closure, illustrating how “cell-interface” engineered liposomes can enhance therapeutic precision.

#### 4.1.2 Stimulus responsive LP

Stimulus responsive drug delivery systems have emerged as a prominent research focus in recent years due to their potential in tailored drug delivery. Since various wounds exhibit distinct microenvironments, these intelligent LPs can react to internal and external stimuli, facilitating targeted drug release to meet the specific requirements of different wounds. For example, the imipenem-loaded gold-coated liposome with LPS-targeting aptamer@hydrogel (ILGA@Gel spray) (Pan et al., 2023) exhibits excellent flow properties, conforming to wound shapes and swiftly transforming into a gel layer for hemostatic purposes upon exposure to near-infrared light. Additionally, ILGA@Gel offers bacterial scavenging and macrophage modulation, among other benefits. Research findings demonstrate that wounds treated with ILGA@Gel display minimal bacterial biofilm and significant wound healing progress, contrasting with visible skin gaps in the control group. This underscores the superior efficacy of ILGA@Gel in infected wounds. Clinically resistant bacteria will invade deeper tissues, and the system is capable of handling deep subcutaneous infections. Furthermore, a pH responsive aqueous liposome has been developed with prolonged blood circulation (Wang D. Y. et al., 2021). Studies show that DCPA-H2O LPs swiftly accumulate at infection sites just 10 min post-tail vein injection, reaching levels ten times higher in the acidic tumor microenvironment compared to the systemic average. This self-targeting ability demonstrates the potential of LPs-DS to target not only infection sites but also tumor sites effectively. For infected skin wounds, a tailored antibiotic delivery system has been developed, utilizing low-temperature-sensitive LPs [LTSL(s)] in conjunction with magnetic resonance-guided high-intensity focused ultrasound thermotherapy to achieve precise delivery. Results indicate that at normal body temperature, LTSL(s) releases ciprofloxacin slowly post intravenous administration, yet controlled warming to 42 °C triggers a four-fold upsurge in drug concentration, significantly enhancing the bactericidal effect on *S. aureus* (Wardlow et al., 2016). We propose that this approach enhances the capacity to regulate and selectively release drugs in infected wounds, with the non-invasive nature of controlling drug release and antibacterial action showing potential for targeted delivery of concentrated antimicrobial agents to wounds. Overall, these intelligent stimulus responsive LPs show great promise for future applications.

#### 4.1.3 Composite LP

Composite LPs are defined as systems formed by combining multiple substances or technologies to create integrated platforms. In this Review, system as a whole, that do not exhibit a clearly defined targeting function or stimulus responsive behavior are classified as Composite LPs that leverage the individual advantages of each component and collectively enhance therapeutic outcomes. By contrast, LPs with explicit targeting effect or well stimulus responsive mechanisms are discussed separately under the corresponding categories. For instance, a liposome-centered hydrogel with Garvicin KS (the GarKS gel) (Thapa et al., 2021) was formulated with optimal viscosity, rheology, and sustained controlled drug release, demonstrating reduced drug resistance rates and superior performance compared to Fucidin cream in animal studies. Other noteworthy creations include catechin-in-cyclodextrin-in-phospholipid liposome (CCPL) (Sinsinwar and Vadivel, 2021) and Vitis vinifera leaf extract liposomal Carbopol gel (VVL-liposomal gel) (Elmaidomy et al., 2023), developed by researchers to exhibit exceptional efficacy against MRSA infections, showcasing significant improvement over traditional LPs. Moreover, a multi-mechanistic antimicrobial approach has been implemented to combat drug resistance, yielding a multifunctional antimicrobial mechanism platform under hypoxic conditions (Wang et al., 2023). This platform combines Cypate-coupled antimicrobial peptides, photothermal therapy (PTT), and photodynamic therapy (PDT) to achieve robust antimicrobial capacity. A comprehensive overview of additional studies is detailed in Table 1.

**TABLE 1:** Summary of LP-based systems for infected wound treatment.

System/Active ingredient	Type	Encapsulation efficiency	Size/Zeta potential	Potential application/Challenges
Macrophage membrane–coated cationic liposomes in MN patch/Vancomycin + BPQDs (Tang et al., 2025)	Targeted LP (macrophage membrane)	92.7%	103.4 nm/−13.9 ± 3.3 mV	Site-specific delivery for drug-resistant skin infections, integrates antibiotic and phototherapy but challenge in translation and manufacturing control
Iron oleate containing lipid nanoparticles (IO-LNPs)/iron oleate (Zhou et al., 2025)	Stimulus responsive LP (ROS responsive)	–	114 ± 2 nm/−12.3 ± 1.73 mV	Antibacterial therapy but ROS control and translation need attention
ILGA@Gel/Imipenem (Pan et al., 2023)	Stimulus responsive LP (light responsive)	33.1%–51.3%	295.3 nm/−18.8 ± 0.7 mV	Treat multidrug-resistant infections, but challenges in large-scale clinical use
Berberine liposome gel/Berberine (Li et al., 2023)	Stimulus responsive LP (temperature responsive)	–	100 nm/−4.39 ± 0.82 mV	Promote biofilm eradication and accelerates wound healing, but face challenges in optimizing drug release and ensuring long-term stability
LTSL(s)/Ciprofloxacin (Wardlow et al., 2016)	Stimulus responsive LP (temperature responsive)	95%	153 ± 1.1 nm/−	Targeting ciprofloxacin delivery but face challenges with fine-tuning drug stability and release in diverse biological conditions
Photo-triggerable LPs (Zhang et al., 2002)	Stimulus responsive LP (light responsive)	–	–	In vitro- a photo-triggered Formation of protein hydrogels in drug delivery
DCPA/Ciprofloxacin (Wang D. Y. et al., 2021)	Stimulus responsive LP (pH responsive)	–	100 nm/−0.2 ∼ +25.2 mV	Self-target to infection sites, but challenges remain in optimizing release kinetics and large-scale production
Collagen gel with liposome (Col III-AC-FL)/Antimicrobial peptide (Wang X. et al., 2023)	Composite LP (Multiple antimicrobial mechanisms)	–	50 nm/−52 mV	Multiple antimicrobial mechanisms, but challenges persist in optimizing the release profile and ensuring stability
Liposome with chitosan/Chlorhexidine (Hemmingsen et al., 2022)	Composite LP (Antimicrobial activity)	70.4% ± 3.9%	393 ± 23 nm/83.3 ± 3.1 mV	Enhance antimicrobial activity, but encounter challenges in large-scale production and stability control
CCPL/Catechin (Sinsinwar and Vadivel, 2021)	Composite LP (Anti-MRSA)	98.9%	206 nm/−40.6 mV	Improve water solubility and antibacterial activity of catechin, but face challenges in long-term stability and controlled release of the encapsulated compound
GarKS gel/GSH+GarKS+EDTA (Thapa et al., 2021)	Composite LP (Physical performance)	–	180 nm/−	Enhance GarKS stability and efficacy, but face challenges in optimizing peptide concentration for prolonged antibacterial effect and ensuring stability
VVL-liposomal gel/Vitis vinifera leaf extract (Elmaidomy et al., 2023)	Composite LP (Anti-MRSA)	88% ± 3%	50–200 µm/−50–63 mV (overall)	Promote wound healing and antibacterial effects against MRSA, but face challenges in ensuring stable, controlled release and optimizing for large-scale production
Chlorella pyrenoidos-based antibiotic liposomal gel/Chlorella pyrenoidosa (CP) (Sun et al., 2025)	Composite LP (biofilm-interfering)	–	100–200 nm/≈ −10 mV	Clinical translation
F-127 hydrogel superstructure loaded with liposomal nanobubbles (NB)/fat extract (Wang et al., 2025)	Composite LP (Antimicrobial property)	–	∼265 nm/15 mV	Treat MR infection-accompanied wounds/Controlled activation
Vesicular phospholipid gel (VPG, highly concentrated liposomes)/Thymol (Keser et al., 2025)	Composite LP	62%	140–150 nm/−5 ∼ −8 mV	Good antibacterial activity
Gelatin Methacryloyl Nanoniosome (Nio-Thymol@GelMa)/Thymol (Moghtaderi et al., 2023)	Composite LP (Antimicrobial property)	72%	184 ± 6 nm/−21 ± 1 mV	*In vitro* -antibacterial efficacy and compatibility *in vitro*
Chitosan nanoniosome (TCH-Nio@CS)/Tetracycline hydrochloride (Pourseif et al., 2023)	Composite LP (Controlled release)	65.0% ± 1.6%	134 ± 8 nm/−21.4 ± 1.3 mV	*In vitro* -a novel drug delivery system to treat bacterial infections

**FIGURE 4:**
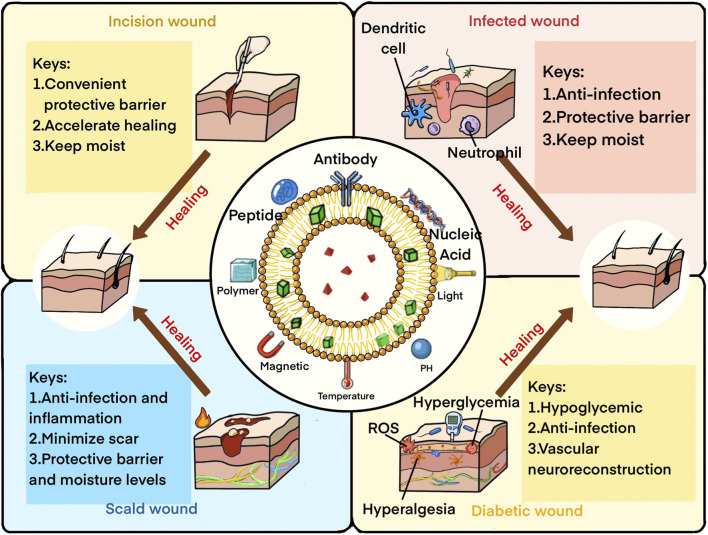
Microenvironment and keys to healing of four types of common skin wound with LP-based systems.

In conclusion, recent research on infected wounds has successfully tackled drug resistance by employing multiple antimicrobial mechanisms. Future endeavors should concentrate on integrating the versatility and targeting proficiency of functionalized LPs-DS with diverse antimicrobial mechanisms. By adopting this approach, optimal treatment outcomes for infected wounds can be achieved, mitigating the risk of drug resistance emergence (Figure 4).

### 4.2 LP-based systems for diabetic wound treatment

Diabetic wounds present a complex challenge marked by dysregulated angiogenesis, neuropathy, prolonged inflammatory responses, heightened levels of reactive oxygen species, and persistent bacterial colonization, rendering them challenging to manage (Burgess et al., 2021). Elevated blood glucose levels impact the function and quantity of cytokines during the inflammatory stage, increasing the risk of wound infection. Additionally, in the proliferative phase, epidermal stem cells and fibroblasts are inhibited, while high blood glucose in the remodeling phase disrupts enzyme and growth factor functions (Patel et al., 2019). Furthermore, challenges in extremity wound healing contribute to the development of diabetic foot complications (Li et al., 2020), attributed to hyperglycemia affecting blood vessel endothelial function, altering hemodynamics, and prompting peripheral neuropathy characterized by reduced pain and temperature sensation, leading to secondary wound complications (Armstrong et al., 2023). Overcoming these obstacles is crucial to establishing a conducive wound healing environment. Future strategies must focus on correcting these negative dynamics, targeting wound abnormalities by regulating blood glucose levels, repairing damaged cells, supplementing with appropriate cytokines to restore cellular function, managing infections, and promoting peripheral nerve regeneration (Chin et al., 2019). Although current treatments center on blood glucose control, blood pressure management, anti-infection measures, and vascular and nerve reconstruction, overall outcomes remain suboptimal. Building on traditional approaches, recent studies have identified macrophages as potential therapeutic targets to enhance diabetic wound healing (Sharifiaghdam et al., 2022). Additionally, researchers have highlighted the significance of oxidative stress and antioxidants in diabetic wound healing (Deng et al., 2021). With the emergence of diverse novel methods, the utilization of LPs-DS has gained traction in this field.

#### 4.2.1 Targeted LP

Researchers have recently introduced a novel strategy that integrates gene therapy and tissue engineering for treating diabetic skin wounds by developing gene-liposome nanocomplexes to serve as a dermal substitute (Wang et al., 2023). These nanocomplexes allow for sustained, targeted delivery of the SDF-1α gene to the wound site, thereby stimulating angiogenesis and facilitating diabetic wound healing. Mechanistically, SDF-1 acts as a chemotactic cue through CXCR4/CXCR7 to recruit CXCR4^+^ reparative cells into the wound bed, activating PI3K/Akt and MAPK/ERK pathways to enhance migration, survival, angiogenesis and re-epithelialization (Ceradini et al., 2004; Bianchi and Mezzapelle, 2020). This biocompatible and biodegradable dermal substitute offers a safe and efficient therapeutic approach. Demonstrating significant efficacy in diabetic rats, this method holds promise for clinical application. With its precise gene and therapeutic agent delivery, promotion of angiogenesis, and accelerated healing, this approach provides a groundbreaking solution to diabetic wounds. Furthermore, an optimized lipoproteoplex (LPP) formulation effectively delivered siRNA targeting Keap1 (Rabbani et al., 2017), rejuvenating the antioxidant capacity of the targeted molecule, restoring the homeostatic environment in diabetic wounds, and expediting wound healing. However, LPP may require further addition of stabilizers and ensuring the structure of the nanoparticles without compromising biosafety as well as off-target effects. This combination of gene therapy and drug delivery opens up new avenues for disease management. Gene therapy stands out as a promising form of targeted therapy, introducing specific nucleic acid sequences to induce gene-specific modifications. A recent study explicitly addressed diabetic-ulcer-associated chronic inflammation and formulation barriers by integrating liposomal encapsulation with a dissolving HA microneedle platform, thereby improving local delivery to the wound bed. This strategy highlights that localized “device-enabled” targeting can also enhance therapeutic precision and reduce off-target exposure in diabetic wound management (Hu et al., 2025).

#### 4.2.2 Stimulus responsive LP

The onset of oxidative stress due to excess ROS prompted researchers to develop a ROS responsive system (Shi et al., 2024). This system not only scavenges surplus ROS to alleviate oxidative stress but also exhibits bactericidal properties for microbial infection control. Furthermore, it suppresses the release of inflammatory factors, modulates the inflammatory response, and enhances angiogenesis, ensuring adequate oxygen and nutrient supply to the wound for effective repair and healing. With rapid *in situ* gel formation and sustained release capabilities tailored to the high ROS diabetic microenvironment, this modified system shows promise in promoting diabetic wound healing significantly. It opens new possibilities in diabetic wound treatment. However, the toxicity issues of silver ions and the complexity of the fabrication process may affect its further widespread use in the future. To validate its efficacy and safety, further investigations are needed, culminating in clinical validation and application. Moreover, we anticipate broader clinical applications of this versatile system across various diseases.

#### 4.2.3 Composite LP

Researchers formulated Tailored Citicoline Chitosan-Coated Liposomes (CT-CS-LPs) by incorporating chitosan into LPs to improve stability and prolong drug release (Eid et al., 2022). The inherent antimicrobial properties of chitosan play a crucial role in treating diabetic wounds. *In vivo* experiments have demonstrated that this system facilitates wound healing by mitigating inflammation, promoting angiogenesis, and enhancing tissue remodeling. The synergistic combination of hydrogels and LPs presents a promising strategy, leveraging the drug storage and skin permeability of LPs alongside the excellent physical attributes of hydrogels (Yu et al., 2021). A Resveratrol-loaded liposomes gel (RV-liposomal gel) (Pandey et al., 2023), developed for treating diabetic foot ulcers in mice, has shown comparable efficacy to ©SILVEREX. Furthermore, significant enhancements in fasting blood glucose levels in mice are attributed to the system’s antioxidant, anti-inflammatory, and hypoglycemic properties. Advancements in nanotechnology have seen the integration of electrostatic spinning technology into drug delivery systems. As a result, the nanocomposite membrane taxifolin liposome centered with polyvinyl alcohol/chitosan (PVA/CS/TL) (Ding J. Y. et al., 2023), known for its remarkable antioxidant and antimicrobial properties, has exhibited a capacity to facilitate wound healing in diabetic mice. Continued research and development showcase the potential of these innovative drug delivery systems as a novel approach in diabetic wound treatment, providing a more effective and sustainable therapeutic option for diabetic patients. Further studies are detailed in Table 2.

**TABLE 2:** Summary of LP-based systems for diabetic wound treatment.

System/Active ingredient	Type	Encapsulation efficiency	Size/Zeta potential	Potential application/Challenges
Gene liposome nanocomplex-loaded dermal substitute (GDS)/Genes (Wang Y. et al., 2023)	Targeted LP (SDF-1a- recovering angiogenesis)	–	96 ± 13 µm/- (overall)	Gene therapy, but face challenges in ensuring consistent gene release and optimizing for human clinical application
LPP/SiRNA (Rabbani et al., 2017)	Targeted LP (Keap-Nrf2)	–	174.22 ± 8.71 nm/34.5 ± 1.7 mV	Gene therapy, but face challenges in optimizing the delivery vehicle for stability and clinical-scale application
Cocktail entrapped liposomal formulation (LCP)/Bacteriophages (Chhibber et al., 2018)	Targeted LP (Phage- *Staphylococcus aureus*)	87%	212 nm/−	Challenges in optimizing phage stability
Macrophage liposome and GelMA microneedle patches with Purpurolide C (PC@MLIP MN)/PC (Liu et al., 2023)	Targeted LP (Targeting TLR4-MD2 dimerization and MYD88 phosphorylation)	5.91% ± 0.57%	≈150 nm/−60 mV	A promising novel biomaterial for the management of diabetic wound
PVA/CS/TL/Taxifolin (Ding Q. et al., 2023)	Targeted-LP (IκBα-NF-κB)	79.17% ± 1.69%	294.12 ± 5.73 nm/−33.16 ± 5.22 mV	Challenges in optimizing drug release and ensuring consistent bioavailability
Phosphatidylserine-modified liposome (PS DexP)/Dexamethasone (Gauthier et al., 2018)	Targeted LP (Influencing macrophage polarization)	–	100–150 nm/−0.81 ± 0.66 mV	In vitro- a promising robust liposome-based platform
Nanocomposite liposome-hydrogel/SDF-1α (Yu et al., 2020)	Targeted LP(SDF-1a-MSCs migration)	88% ± 6%	236 ± 3 nm/−48 ∼ −41 mV	In vitro- a promising theoretical protein delivery system
Pluronic F127 liposome/Curcumin (Zhou et al., 2023)	Targeted LP (Nrf2-Keap1)	–	–	In vitro- may contribute to wound healing
Liposome-mediated HA microneedle patch/β-elemene (Hu et al., 2025)	Targeted (site-specific delivery)	91.67% ± 1.25%	94.36 nm/−	Addresses chronic inflammation/fabrication complexity
Curcumin liposomes and silver nanoparticles centered with hyaluronic acid hydrogel (HA@Cur@Ag hydrogel)/Curcumin (Shi et al., 2024)	Stimulus response LP (ROS responsive)	85.99% ± 1.362%	102.033 ± 1.097 nm/−2.517 mV	A multifunctional injectable system but face challenges in optimizing sustained release and ensuring long-term stability
GelMA - lipoSDF/SDF-1α (Yu et al., 2021)	Composite LP (Immunoregulation)	–	–	Promote macrophage phenotype changes and skin tissue regeneration,but face challenges in optimizing long-term drug release and clinical-scale application
Cationic elastic LPs/EGF, IGF-I, PDGF-A (Choi et al., 2017)	Composite LP (Great permeation)	80%	107 ± 0.757 nm/56.5 ± 1.13 mV	Improve growth factor permeation with cationic elastic liposomes, but face challenges in consistency and long-term stability for clinical use
Deformable LPs-In-Chitosan Hydrogel/Curcumin (Ternullo et al., 2019)	Composite LP (Multi-property)	84.1% ± 6.1%	322.7 ± 12.5 nm/−47.7 ± 5.8 mV	In vitro- show some different and promising properties
Transfersome with cellulose/hyaluronate/Anthocyanins and Ellagitannins (Castangia et al., 2021)	Composite LP (Oxidative stress regulation)	95% ± 3%	95 ± 4 nm/−19 ± 2 mV	In vitro- a promising system for skin wounds with oxidative stress
Nanofibers with Embedded Liposomes (BHA-SIM-LIPO-NF)/Simvastatin (Casula et al., 2023)	Composite LP (Immunoregulation)	80% ± 4%	105.9 ± 4.4 nm/−18 ± 3 mV	In vitro- a promising efficient dressing for chronic wound
Polycaprolactone/Gelatin Nanofiber Membranes Containing EGCG-Loaded Liposomes (PCL-EGCG-PEG)/EGCG (Pires et al., 2019)	Composite LP (Antioxidant activity)	82.4% ± 1.9%	114 ± 22 nm/−	In vitro- a Promising system for the prevention or treatment of skin diseases
Hyalutransfersome/Oleuropein and lentisk oil (Sklenarova et al., 2023)	Composite LP (Oxidative stress regulation)	68% ± 2%	101 ± 1 nm/−67 mV	In vitro- a Promising system for the prevention or treatment of skin diseases
10argan-hyalurosomes (Manca et al., 2021)	Composite LP (Oxidative stress regulation)	65% ± 7%	140 ± 8 nm/−73 ± 5 mV	In vitro- a natural alternative
CT-CS-LPs/Citicoline (Eid et al., 2022)	Composite LP (Sustained-release)	50.7%	211.6 nm/32.1 mV	Sustained-release properties, but face challenges in human diabetic patients
RV-liposomal Gel/Resveratrol (Pandey et al., 2023)	Composite LP (Oxidative stress regulation)	85.32% ± 1.24%	134.34 ± 9 nm/−28 ± 0.25 mV	Reduce oxidative stress, but face challenges with ensuring sustained release and optimizing for clinical use
Chitosan-liposome (SP-CH-LP)/Chlorhexidine (Mengoni et al., 2017)	Composite LP (Treat difficult wounds)	66% ± 3.5%	243 ± 24 nm/32 ± 1.0 mV	In vitro- a novel way to treat difficult wounds
Nanohyaluronan-glycerosomes/Curcumin (García et al., 2025)	Composite LP (enhanced angiogenesis)	80% ± 9%	543 ± 39 nm/−59 ± 5 mV	Enhanced angiogenesis
Hydrogen sulfide sustained-release nanospheres (Hou et al., 2025)	Composite LP (sustained-release)	89.51%	130 ± 4.36 nm/33.08 ± 2.96 mV	Regulates inflammatory microenvironment

Treating diabetic wounds presents significant challenges, necessitating optimized LPs-DS that address not only surface wound healing but also microenvironmental changes induced by diabetes. This comprehensive approach aims to address both symptoms and underlying causes, alongside the integration of diverse novel therapies, offering considerable promise for effective diabetic wound management (Figure 4).

### 4.3 LP-based systems for burn wound treatment

Burns result from skin damage induced by heat, radiation, electricity, or chemicals, requiring urgent clinical treatment for severe complications. Infection poses a significant threat to burn victims due to their compromised skin barrier, emphasizing the critical importance of prompt wound coverage and infection prevention (Shpichka et al., 2019). Slow wound healing, infections, pain, and scarring stand as the foremost challenges in burn management (Wang et al., 2018). Typically, burned skin exhibits three distinct zones: a central necrotic zone, a restorable peripheral zone, and a restorable zone with hemorrhage (Nisanci et al., 2010). While early removal of necrotic tissue and autologous skin grafting are primary burn treatment methods, preserving function and aesthetics has gained importance in modern medicine. Scholars emphasize that the ultimate goal of burn treatment is not only physical restoration but also the achievement of aesthetically pleasing outcomes and psychological wellbeing (Schiestl et al., 2013). Growth factors are pivotal in burn healing (Oryan et al., 2017), coordinating wound healing with keratinocytes (Curran and Ghahary, 2013). Anti-inflammatory measures are crucial for burn recovery, as misregulated inflammatory pathways can lead to excessive scarring (Shih et al., 2010). Additionally, nutrition and fluid resuscitation significantly impact burn healing outcomes. Adequate nutritional support is vital for severe burns to counteract hypermetabolism (Rowan et al., 2015). Skin grafts remain the mainstay treatment for extensive burns, while topical drug delivery system dressings are commonly used for daily small skin burns. These dressings should be user-friendly, provide effective barrier coverage, maintain wound moisture, possess anti-inflammatory and antibacterial properties, and promote wound healing by delivering bioactive substances to optimize the wound microenvironment and reduce scarring.

#### 4.3.1 Targeted LP

Anti-Gal, the most prevalent natural antibody in humans, accounting for approximately 1% of immunoglobulins (Galili, 2013), was utilized by the researchers to develop the α-gal liposome (Galili et al., 2010). This liposome features a specific binding epitope that targets the anti-Gal antibody, swiftly recruiting neutrophils and macrophages and stimulating cytokine secretion essential for burn healing. Encouraging results have been obtained in mouse animal experiments, suggesting promising prospects for skin burn treatment due to the abundant presence of anti-Gal in humans. However, further testing in larger animal models and eventual human clinical trials is still required to ensure efficacy and safety across different species and environments. At the same time, the preparation of the a-gal liposome involves the use of rabbit erythrocyte membranes, which may pose challenges in terms of scalability and consistency for large-scale production, and non-animal-derived methods of preparation may be able to avoid potential problems with ethical and regulatory issues associated with animal use. Combining Hematoporphyrin monomethyl ether (HMME) with photodynamic therapy for bacterial infections has proven effective. Encapsulation of HMME with cationic LPs enhances its positive electropositivity and hydrophobic properties, bolstering therapeutic efficacy. Similarly, polymyxin B (PMB) exhibits targeted action against Gram-negative bacteria by binding to their negatively charged phosphate groups. Building upon this, the researchers developed PMB-targeted liposomal photosensitizer (HMME@Lipo-PMB) (Cui et al., 2024), showing promise in tissue repair by modulating macrophage polarization and governing the inflammatory response. This innovative approach promotes acute inflammation in the early stages while mitigating chronic inflammation in later stages, indicating transformative potential for therapeutic interventions. The system is not only targeted, but also has the dual efficacy of clearing infections and mobilizing macrophages, in addition to reducing the resistance problem caused by traditional antimicrobial drugs. However, we still hope that its targeting properties can target a wider range of bacteria, and it is still doubtful whether deep tissue wounds can receive light, and the simplification of the synthesis process may expand the scope of subsequent research and further application.

#### 4.3.2 Stimulus responsive LP

Hydroxymacrogolide (MA) has been recognized for its wound-healing properties and scar reduction capabilities. However, when used in isolation, its permeability is notably limited, and traditional liposome formulations struggle to maintain optimal adhesion to wounds (Hou et al., 2016). Addressing this challenge, researchers developed a Madecassoside liposome with poly (ethylene glycol -ε-caprolactone- ethylene glycol) (PECE-modified MA liposome) (Liu et al., 2020). This innovative formulation exhibits remarkable temperature responsive properties, transitioning from solution to gel and swiftly adhering to the wounded surface at body temperature. Significantly improving adhesion, this liposome demonstrated exceptional wound contraction effects in second-degree burn experiments in rats. Moreover, it effectively exhibited early-stage anti-inflammatory effects, aligning with the strategy of reducing wound area promptly to minimize scar formation during the healing process. The drug loading problem may need to be improved by optimizing the preparation process, while the system’s resistance to infection has not been suggested since burns are usually accompanied by infections as well, but its temperature responsive properties are particularly prominent. Envisioned as an *in situ* gel for sustained drug delivery, this LPs-DS holds promise for future applications in continuous wound therapy.

#### 4.3.3 Composite LP

The introduction of LPs with a Silk Fibroin Hydrogel Core (Xu et al., 2017) led to a nearly doubled wound healing rate on day 14 compared to the conventional LPs group. Researchers attribute this success to the system’s significant promotion of cell proliferation and stimulation of angiogenesis. A novel formulation, liposome with gel matrix loading pentoxifylline-valsartan (PTX-VAL-LG), offers a slow sustained drug release mechanism (El-Salamouni et al., 2022), accelerating burn wound healing by targeting the HMGB-1/TLR signal pathway, showcasing the potential for early burn treatment strategies. In a study involving pigs, a liposome-gelatin membrane system (Nunes et al., 2016) was compared with Silver Sulfadiazine Ointment and duoDerme® dressing, revealing comparable granulation tissue development and superior scar repair effectiveness, instilling confidence in the future application of this LPs-DS. To enhance drug penetration into the skin, LPs were optimized through the addition of penetration enhancers like laurocapam inserted into the liposome membrane (Xu et al., 2018) or propylene glycol-modified LPs (Kianvash et al., 2017), both demonstrating improved efficacy in promoting skin regeneration and reducing inflammation. Photodynamic Antimicrobial Chemotherapy (PACT) proves to be a promising approach to combat multidrug-resistant bacteria (Jiang et al., 2016). CDL2-PACT (Mai et al., 2021), a cationic liposome-based drug delivery system for Gram-negative bacteria, generates localized and transient ROS (about 7 times that of the control group), which induce the membrane integrity of bacteria is compromised, thereby enhancing antibacterial efficacy and facilitating wound healing, offering the potential for overcoming Gram-negative bacteria drug resistance. Continued progress and research in this field are detailed in Table 3.

**TABLE 3:** Summary of LP-based systems for burn wound treatment.

System/Active ingredient	Type	Encapsulation efficiency	Size/Zeta potential	Potential application/Challenges
α-gal liposome (Galili et al., 2010)	Targeted LP (Targeting the anti-Gal antibody)	–	0.5–15 μm/−	Optimization could focus on refining the liposome composition for better efficiency and addressing potential immune system limitations
Total flavonoids composite phospholipids liposome gel (TFOFB-CPLG)/Oxytropis falcata Bunge (Zeng et al., 2020)	Targeted LP (p38 MAPK,NF-kB/IkB)	59.15% ± 2.4%	210.8 ± 12.1 nm/−31.2 ± 3.5 mV	Could focus on optimizing the release rates and enhance the liposomal stability for even longer-term efficacy
Deformable liposomal ointment (TRA DLs and EGF CDLs)/TRA/EGF (Lu et al., 2019)	Targeted LP (EGFR and HB-EGF)	63.73% ± 4.48%	16.00 ± 1.01 nm/−	Challenges on refining the drug release profiles and improving the formulation for broader clinical application
HMME@Lipo-PMB/PMB (Cui et al., 2024)	Targeted LP (Modulating macrophage polarization)	82.12%	98.64 ± 0.914 nm/29.93 ± 0.914 mV	Need to improve targeting efficiency and minimize side effects on surrounding tissues
PECE-modified MA liposome/Madecassoside (Liu et al., 2020)	Stimulus response LP (Temperature responsive)	68.26% ± 2.46%	213.43 ± 4.68 nm/−23.80 ± 15.37 mV	Temperature responsive, excellent wound adhesion and sustained release, though further optimization is needed for stability and release control
PTX-VAL-LG/Pentoxifylline/Valsartan (El-Salamouni et al., 2022)	Composite LP (Sustained release)	28.8%/86.0%	179 nm/130 nm	Good drug embedding efficiency, sustained release characteristics, and target inflammatory pathways, but still need to address issues of liposome stability and drug release control
Ethosomal Gel (Tβ-4 EP gel)/Thymosin β-4 (Fu et al., 2019)	Composite LP (Great permeation)	63.2% ± 4.5%	127.8 ± 3.2 nm/25.1 ± 2.8 mV	Enhanced drug permeation and faster wound healing, but challenges remain in optimizing the gel’s long-term stability
Cationic lipid composition of lipo-Sinoporphyrin sodium (CDL2-PACT)/DVDMS (Mai et al., 2021)	Composite LP (Photodynamic eradication)	–	122.4 nm/40.2 mV	Optimize liposomal formulation and improving drug loading efficiency
Propylene glycol nanoliposomes (Cur-PgL)/Curcumin (Kianvash et al., 2017)	Composite LP (Early healing)	84.66% ± 2.4%	147 ± 6 nm/−28.53 ± 0.709 mV	Early stage healing properties, but could focus on enhancing the formulation’s antibacterial strength
Liposome with Silk Fibroin Hydrogel/bFGF (Xu et al., 2017)	Composite LP (Early healing)	100% ± 4.3%	99.8 ± 0.5 nm/−9.41 ± 0.10 mV	Accelerate the wound closure with deep second degree scald but challenges in optimizing sustained release and large-scale clinical application
Liposome with Silk Fibroin Hydrogel/bFGF (Xu et al., 2018)	Composite LP (Hair follicle growth)	85%	103.3 ± 0.2 nm/−2.31 ± 0.05 mV	The hair growth and hair follicle are obviously improved but face ensuring effective long-term stability
Liposome with Gelatin-based membrane/Usnic acid (Nunes et al., 2016)	Composite LP (Pig model and great permeation)	≈100%	–	Better healing in Pigs but face challenges in optimizing skin permeation and release kinetics for broader clinical use
Liposome with chitosan gel/EGF (Degim et al., 2011)	Composite LP (Sustainable release)	58.1%	4.44 μm/−	Stable long retained and safely used in burns but face challenges in optimizing sustained release and stability for extended use
Chlorhexidine-loaded lipid and chitosan-based nanocarriers with chitosan hydrogel (HG-CHI-CHX)/Chlorhexidine (Hemmingsen et al., 2021)	Composite LP (Infection prevention for acute wound)	74% ± 2%	≈202.73 nm/79.0 ± 3.7 mV	In vitro- A suitable formulation in acute wounds
Liposomal hydrogel with povidone-iodine (PVP-ILH)/Povidone-Iodine (Beukelman et al., 2008)	Composite LP (Anti-inflammation)	–	–	In vitro- A beneficial and pronounced anti-inflammatory effect
Liposomal bFGF combined with injectable hydrogel/bFGF (Tu et al., 2025)	Composite LP (sustained local release)	98.6%	85.30 ± 14.09 nm/−21.7 ± 2.07 mV	Enable sustained local release; promotes angiogenesis, collagen deposition, and re-epithelialization

It is our belief that LPs-DS hold significant promise in facilitating skin burn healing. Future endeavors will focus on perfecting formulations that yield LPs-DS with optimal coverage, anti-inflammatory, antibacterial properties, and the ability to create a conducive wound microenvironment for enhanced healing outcomes (Figure 4).

### 4.4 LP-based systems for incision or excision wound treatment

Incision or excision wounds are among the most common types of acute skin injuries with a typical healing process. Incision wounds are often caused by everyday sharp objects, resulting in neater edges, while the depth and length of the wound vary based on the nature and extent of the trauma, involving less damage to vital structures like blood vessels and nerves. In contrast, excision wounds typically occur during surgical tissue removal, presenting with regular or irregular shapes and varying depths based on the tissues removed. Incision and excision wounds are commonly used to simulate real-life skin injuries by cutting or removing skin with medical instruments (Ansell et al., 2014). ADDIN While wounds from medical interventions are usually surgically closed, smaller daily-life injuries are typically managed with bandages to prevent infection, necessitating regular dressing changes. However, as exudate dries and the wound heals, the dressing may adhere, risking damage to new tissue during changes, causing pain and impeding healing (Dhivya et al., 2015). Optimal wound dressings should maintain a moist, sterile environment (Qi et al., 2022), offering physical protection and fostering tissue regeneration to expedite the healing process and allow for easy changing.

#### 4.4.1 Targeted LP

The α-gal liposome has demonstrated efficacy in treating diabetic wounds and incision wounds (Wigglesworth et al., 2011), accelerating wound healing and reducing scar formation. This has led to anticipated clinical applications. Firstly, as anti-Gal is abundant in immunocompetent populations, the α-gal liposome’s specific binding to this antibody triggers a subsequent immune response. Considering the known role of macrophages in wound recovery, the activation of macrophages through the α-gal liposome is more straightforward compared to the alternative of injecting activated macrophages directly into the wound. This method eliminates the need for *in vitro* culturing of macrophages, offering convenience and high efficacy, thus showing promising potential for skin wound treatment. Similarly, the limitations of the α-gal liposome have been discussed previously.

#### 4.4.2 Composite LP

Researchers developed multifaceted lyophilized liposomal wafers (Avachat and Takudage, 2018) to create a moist wound bed upon contact with wound exudate. This innovative design enables the wafer to maintain drug localization, concentration stability, and robustness, establishing it as a superior drug delivery system. Verification through wound healing experiments in rats further validated its efficacy in promoting epithelialization. Another noteworthy development is the Film-Forming Spray (Umar et al., 2021), engineered for easy application and uniform distribution, forming a protective film matching the wound texture to hasten scab formation, effectively preventing blood loss and infection. *In vivo* studies demonstrated favorable wound healing outcomes, absence of residual scarring, and regrowth of hair in the scarred area, highlighting its convenience and effectiveness as a carrier for wound healing drugs. Ethanol serves as a penetration enhancer by altering the cellular arrangement in the lipid layer of the stratum corneum, thereby decreasing its structural density (Wang et al., 2007). To capitalize on this effect, researchers paired the ethosomal system, tailored to incorporate ethanol (Magdy et al., 2022), with Mebo® ointment for wound treatment in mice. Encouragingly, this approach showed a significant enhancement in wound healing and complete re-epithelialization of the dermis, highlighting its potential for drug delivery. Furthermore, in a study involving large animals such as dogs, the niosomal system (Ali et al., 2018) was compared with Panthenol® 2% cream, demonstrating a notable reduction in the inflammatory phase of the wound and an earlier onset of the proliferative phase. Detailed results of the study on LPs-DS for this type of wound are presented in Table 4.

**TABLE 4:** Summary of LP-based systems for incision/excision wound treatment.

System/Active ingredient	Type	Encapsulation efficiency	Size/Zeta potential	Potential application/Challenges
α-gal Liposome (Wigglesworth et al., 2011)	Targeted LP (Targeting the anti-Gal antibody)	–	–	Address potential immune system limitations
Artificial hair follicles seeding hydrogel (AHFS)/Tideglusib, Tamibarotene (Ji et al., 2024)	Targeted LP (Activating the PI3K/AKT)	92.8% ± 2.9%/85.9% ± 2.2%	87.7 ± 1.23 nm/−	A novel perspective in biomaterial design for scarless wound healing and functional skin restoration
Foslip-PDT (Garrier et al., 2011)	Stimulus responsive LP (Light responsive)	–	–	Combination of photodynamic therapy and drug delivery systems but suggest further investigation into the mechanisms of its action
Liposome centered with Film-Forming Spray of chitosan (FFSWSC)/hEGF (Umar et al., 2021)	Composite LP (Controlled release)	99.87%	219.3 nm/−39.7 mV	Could refine the controlled release properties and enhance the consistency of the film-forming properties for industrial use
Lyophilised liposomal Wafers/Gatifloxacin (Avachat and Takudage, 2018)	Composite LP (Drug loading)	78.33%	120.5 nm/−	Refine the release profile and drug loading efficiency
Niosomal gel/*Hypericum perforatum* (Ali et al., 2018)	Composite LP (Dog model and sustainable release)	80%	490.45 ± 27.64 nm/−41.10 ± 0.84 mV	Better healing in Dogs and could focus on the long-term stability of the niosomal gel and its reproducibility for broader clinical applications
Atrosome-2/Atorvastatin (Abootorabi et al., 2022)	Composite LP (Sustainable release)	86.15% ± 0.58%	196.33 ± 6.45 nm/−20.73 ± 0.98 mV	Sustained drug release and stability need to be improved
Liposomes and penetration enhancer-containing vesicles (PEG-PEV)/Curcumin (Castangia et al., 2014)	Composite LP (Anti-inflammation)	68% ± 7%	188 ± 9 nm/−11 ± 3 mV	Strong anti-inflammatory effects but challenges in drug loading efficiency and stability
Methylene blue (MB) niosome/MB (Farmoudeh et al., 2020)	Composite LP (Great physical property)	63.27%	147.8 nm/−18.0 mV	Could focus on improving stability and drug loading
Chitosan liposomal hydrogel/Ricinoleic acid (Nada et al., 2018)	Composite LP (Great physical property)	90–95%	42–175 μm	Could focus on refining the stability and cytotoxicity issues of higher RA concentrations
Nanofibrous mats/Cefazolin (Mansouri et al., 2022)	Composite LP (Great physical property)	–	184 ± 8 nm/−	Optimize the mechanical properties and further enhance the drug release profile could improve its clinical applicability
Anthocyanin complex (AC) noisome gel/AC (Priprem et al., 2018)	Composite LP (Sustainable release)	–	167.8 nm/−	Could focus on sustained release and its mucoadhesive properties
Oligochitosan nanoparticle complex (OCH-Lip)/Curcumin (Nguyen et al., 2019)	Composite LP (Scar treatment)	91.7%	282.7 nm/23.7 mV	A strong candidate for commercial development as a wound healing and scar treatment formulation
Quercetin loaded multiphase hydrogel (QLH)/Quercetin (Jangde et al., 2018)	Composite LP (Sustainable release)	–	–	Could enhance the drug release profile and stability for better long-term use
Ethosomal gel/Metformin (Magdy et al., 2022)	Composite LP (Sustainable release)	55.3% ± 0.07%	5,770 ± 179 nm/9.07 ± 0.2 mV	Could focus on refining the encapsulation efficiency and improving the long-term stability of for broader clinical applications
Liposomal hydrogel/bFGF (Geng et al., 2025)	Composite LP (sustained release)	–	192 nm/−42.4 mV	Good healing effect/challenge in sustained release
Transfersomal delivery system/*Centella asiatica* extract (Lapmanee et al., 2025)	Composite LP (skin penetration)	70%/90%	135.22 ± 4.80 nm/−26.13 ± 0.58 mV	Great skin penetration/challenge in formulation stability
Niosome-based hydrogel/Quince extract (Ebrahimnejad et al., 2025)	Composite LP (sustained release)	34% ± 1.03%	197.2 ± 0.21 nm/−6.24 ± 0.09 mV	Sustained release

Enhancing the barrier function and replaceability of LPs-DS with inherent anti-inflammatory and antibacterial properties holds the key to expediting wound healing for this common type of acute cut wound (Figure 4).

### 4.5 LP-based systems for other wound treatment

Various types of skin wounds, including frostbite and pressure sores, necessitate careful selection of appropriate dressings. LPs-DS have shown promise in treating these varied wounds.

Local hypoxia can significantly impede tissue survival and wound healing, hence the development of artificial oxygen carriers as a novel therapeutic approach. One such innovation is the design of left-shifted liposomal hemoglobin vesicles (HbVs) (Plock et al., 2009). In a study involving mice with skin flap ischemia, the survival rate of flap tissue increased from 33% in the control group to 57% after the injection of HbVs. The researchers proposed that this system enhanced the survival of severely ischemic wound edges by promoting neovascularization. The method of increasing oxygen saturation is a brand-new idea to solve the hypoxia of deep tissues. However, the fineness of its regulation process and the regulation mechanism need to be further investigated. In addition, whether a large amount of exogenous HbVs will cause an immune response needs to be further observed. Meanwhile, the complexity of its production process as well as the cost control and other issues need to be considered. Flap grafting is often necessary for large skin defects, particularly in the early stages where lack of adequate blood supply hinders flap growth. A hydrogel capable of locally releasing Ag+ and MF-Lip was formulated for this purpose (Mao et al., 2019). By using the properties of the liposome as a reservoir and delivery carrier, in combination with the stability of the hydrogel, the necrosis rate of rat flaps was reduced by two-thirds. The multifunctional carrier, noted for its injectability and slow-release function, facilitated angiogenesis by modulating the Bax/Bcl-2/Caspase-3 pathway to decrease flap inflammation and infections. This ultimately led to significantly improved flap survival rates. The future should be devoted to ensuring that silver ions are released at the desired time and site by designing more precise release control systems. It is also important to avoid their overaccumulation which could lead to toxicity and excessive inflammatory responses. An innovative nano-spray was developed to address acute frostbite (Vaghasiya et al., 2019), demonstrating superior effectiveness to silver sulfadiazine in animal models. The enhanced healing efficacy was attributed to the local modulation of blood circulation by cytokines. Pressure ulcers, common among the aging population and individuals with sedentary lifestyles, present further challenges. Researchers formulated PSLs that proved effective in preventing and repairing pressure ulcers by promoting macrophage M1-M2 polarization in mice. Given the pivotal role of macrophages in inflammation control and tissue repair homeostasis (Kusnadi et al., 2019), the strategic manipulation of M1-M2 polarization could potentially extend the application of this approach to various tissue repair scenarios. The ongoing advancement of LPs-DS holds promise for broader applications across different types of wounds.

## 5 Clinical application of LP-based systems

The clinical application of LPs-DS has undergone extensive research, showing significant progress in the field of skin wound healing. While these advancements have not fully aligned with clinical requirements, several studies have successfully transitioned into clinical trials, yielding promising outcomes. Mitigating postoperative skin scarring poses a complex challenge, characterized by the excessive formation of fibrous tissue during the healing process, often exhibiting substantial individual variations. In a prospective double-blind study assessing scarring post-caesarean section, researchers observed enhanced patient satisfaction levels following the application of 8% Trinitest liposomal gel to 20 women (Kohavi et al., 2017). Similarly, a separate double-blind trial involving 40 volunteers in a split-face study demonstrated the efficacy of tranilast 8% gel in preventing and treating acne scarring, leading to reduced scarring, improved skin texture, and enhanced appearance (Weinstein et al., 2020). Concurrently, a single-center, randomized, open Phase II trial involving 36 patients who underwent reticulated skin grafts following burn or reconstructive surgery compared the use of a novel polyvinylpyrrolidone iodine liposomal hydrogel to chlorhexidine gauze. Preliminary results favored the former, providing superior wound surface moisture, faster epithelialization, and improved healing compared to conventional chlorhexidine gauze (Vogt et al., 2001). Another randomized controlled Phase II trial, involving 14 eligible patients with post-reticulated skin graft infections, demonstrated the efficacy of PVP-ILH in managing infected wounds (Vogt et al., 2017). Furthermore, a study with 167 patients suffering from graft wounds investigated the impact of PVP-I liposomal hydrogel (Repithel) on stratified skin grafts, revealing its ability to enhance the healing of reticulated grafts and minimize the risk of graft loss (Hauser et al., 2006). Notably, insulin, known for its proficient wound healing properties and cost-effectiveness relative to other growth factors (Joseph, 1930), was evaluated in the form of insulin liposomal chitosan gel. Clinical findings displayed a notable reduction in erythema and accelerated wound healing time, with a remarkable 16-fold increase in the healing rate compared to the control group (Dawoud et al., 2019). In a randomized controlled study comprising 43 burn patients (Homann et al., 2007), researchers conducted an intra-individual comparison between traditional silver sulfadiazine cream and liposomal PVP-I hydrogel, concluding that the latter significantly expedited the complete healing of burn wounds in comparison to silver sulfadiazine cream. In this regard, we will learn from the clinical translational experience of the above results, with a view to providing ideas for translating the novel liposome-based delivery systems discussed above from the experimental stage to clinical applications. First, tranilast 8% gel (Kohavi et al., 2017; Weinstein et al., 2020) is a PLO-based delivery system that combines components such as the temperature responsive polymer Pluronic F127 and lecithin to ensure efficient penetration and sustained release of the drug. Its temperature responsive property enables it to form a stable gel at body temperature, thus enhancing the local drug concentration and improving the therapeutic effect; its components have good biocompatibility with the skin and low toxicity, which has been verified by animal experiments and preclinical studies to be less irritating to the skin and suitable for long-term use. Subsequent process amplification and standardized production together contributed to the successful conversion to clinical application. Second, The liposome PVP-I complex (Vogt et al., 2001) is a typical test of combining liposomes with iodine-containing dressings. Over the years, iodine-containing dressings (PVP-I) have been shown to be useful in a variety of infected wounds. Unfortunately, PVP-I preparations have been shown to desiccate the wound surface (Homann et al., 2007). Moist treatment of wounds has been shown to improve epithelialization (Vogt et al., 2001), and the incorporation of liposomes enhances the water-locking ability of the dressing, which is perfectly utilized. Meanwhile, in the preparation process, the liposomes can be prepared by adding appropriate concentration of PVP-I to the raw material and mixing, which is more stable and easier to achieve, and can maintain the consistency of the product in the subsequent process of scaling up. The addition of liposomes not only improves the antimicrobial effect and skin compatibility of PVP-I dressings, but also expands its application in clinical trauma treatment. To summarize, in order to address the shortcomings of clinical treatments, selecting materials that have been widely used to compensate for the shortcomings and fusing them with each other, a new type of fast-acting material can be discovered with a simpler step of adjusting the ratio, which is an important point I mentioned in the previous advantages, and at the same time, the discovery of new materials can be applied to a wider variety of contexts, which will not only have clinical effects but also a wider range of research value. In fact, the benefits will be more than we can imagine. Finally, let us turn our attention to insulin liposome chitosan gels (Dawoud et al., 2019), where the concept of Quality by design (QbD), which aims to ensure the quality of the product through a systematic design and optimization process that emphasizes the consideration of quality criteria from the product design stage, has been effectively applied. During clinical translation, QbD helps to identify and control key parameters of material safety and efficacy, such as purity, particle size, and morphology, thereby reducing the risk of clinical trial failure. The stability and reproducibility of the material is ensured through the design space, further guaranteeing its quality consistency in mass production. Ultimately, QbD facilitates safer, controllable and sustainable product development in the clinical application of materials, helping to achieve higher patient satisfaction and compliance with regulatory agencies.

A search on ClinicalTrials.gov using the keywords “skin” and “liposome” retrieved 229 results (as of Novermber 29, 2025). Among these, only a few trials focused on traditional liposome drug treatments, while there was a notable scarcity of research regarding the potential of LPs-DS in skin wound healing. Conversely, there has been a surge in trials exploring the use of polyethylene glycolated modified LPs in oncology, notably in the treatment of cancer. Notably, polyethylene glycol liposomal doxorubicin (PLD) stands out as the first nanomedicine sanctioned for cancer therapy (Barenholz, 2012). While initial studies on PLD showcased promising preclinical outcomes, its clinical application has been somewhat limited. In theory, PLD offers prolonged circulation time, enhanced drug loading stability, optimal permeability to tumor blood vessels, and a buffering effect on drug-related side effects. Despite its approval for treating breast and ovarian cancers due to its potent anti-tumor properties, PLD has not supplanted conventional drug-only therapies. This is primarily due to challenges such as limited drug retention impact within human cancers, interactions between liposomal drugs and the immune system hampering PLD’s therapeutic benefits, and potential variations in drug release rates between humans and mice (Gabizon et al., 2016). The use of polyethylene glycolated LPs has shown promise in mitigating side effects, enhancing plasma stability, and extending circulation time, prerequisites for effective tumor targeting (Kontogiannopoulos et al., 2014), which could also be beneficial in the context of skin wound healing. While oncology remains a prominent research domain, the adaptability of LPs-DS to the intricate tumor microenvironment suggests their potential applicability in skin wound healing. The significant enhancements that LPs-DS can bring to the skin wound healing process underscore their promising role in this field.

## 6 Future perspectives and conclusion

LPs have secured a substantial market presence owing to their favorable biocompatibility and drug-carrying capabilities. To overcome the limitations of traditional LPs, diverse materials and technologies are combined with LPs to harness their individual strengths and synergies, aiming to create a multifunctional liposome delivery system resembling a multi-stage rocket with diverse functionalities. Despite the growing emergence of various formulations of LPs-DS in the field of skin wound healing, significant challenges persist. Challenges facing LPs-DS: (1) Formulation and technology: The presence of individual variations and varying *in vivo* microenvironments further complicates the design of LPs-DS, posing challenges in establishing optimal components. This highlights the ongoing challenge of defining a standard criterion for determining the most effective formulations. In this review, we have categorized different types of skin wounds and endeavored to tailor LPs-DS with specific properties pertinent to each wound type by targeting the wound microenvironment. This approach offers valuable insights for future research endeavors. Moving forward, it is imperative to build upon this groundwork to conduct comprehensive comparative and specific investigations into the optimal formulation of LPs-DS for treating distinct wounds. Although novel composites have shown great potential in experimental studies, their complex composition and preparation process make their scalability for clinical applications limited. In contrast, combining and formulating simple and safe commercialized materials is much more feasible, not only in terms of lower production costs but also in terms of preparation efficiency. The use of existing and proven low-cost materials can simplify the preparation process and reduce potential side effects while ensuring therapeutic efficacy through rational formulation design. In addition, the compounding of simple materials not only reduces technical barriers, but also improves clinical applicability and patient compliance. Therefore, from the perspective of practical application, the use of simple and sustainable raw materials for compounding may be a better strategy to realize the universal application of drug delivery systems. (2) The safety of LPs-DS is a point of concern: While traditional LPs are renowned for their low toxicity, the original premise of this review aimed to leverage the established safety profile of traditional LPs in promoting the swift clinical research and application of LPs-DS. However, the incorporation of diverse materials and technologies in LPs-DS has raised uncertainties regarding their toxicity, with instances of potential harm to normal cells attributed to ionic LPs (Wang et al., 2024). Although some researchers have proposed solutions to mitigate this issue (Shi and Claridge, 2021), this represents just one approach amidst a myriad of enhancements in liposomal formulations. Consequently, a strategic approach involving a thorough examination of the safety considerations, building upon the previously discussed point (1), is imperative to navigate these challenges effectively. Looking at the delivery systems that have been subjected to clinical trials, we find that most scholars are prioritizing those safe materials that have been clinically validated using lower doses for material compounding, in addition to those that have undergone surface modifications, such as liposome surfaces that can be modified by means of PEGylation, etc. In addition, we believe that conducting long-term toxicity experiments on the materials is conducive to a better argument for the safety of the materials, and at the same time We believe that the long-term toxicity of the materials will help to better demonstrate the safety of the materials, and at the same time, we can focus on the novel targeted drug therapy and the combination of therapies with different safe therapeutic modalities, such as thermotherapy mentioned in the article; (3) Advancing clinical research: As mentioned earlier, the application of modified liposomes has been mainly in oncology, and there is a lack of research in the field of skin wound healing, but given the ability of the system to target the complex tumor microenvironment, we believe that it is only a matter of time before it can be applied to the field of skin wound healing. Although many current drug delivery systems have demonstrated excellent performance in experimental studies and achieved significant results in animal experiments, these innovations often remain in the laboratory and are not effectively translated into clinical applications. This phenomenon is due to the limitations of technology, materials and manufacturing processes, as well as regulatory, economic and patient adaptation challenges. In order to promote these research results to the clinic, we need to strengthen preclinical research on the basis of basic research, pay attention to actual clinical needs, optimize the production process, and conduct more extensive clinical trials. Importantly, researchers should not only pursue the publication of academic papers, but also focus on the greater social value of improving patient health and clinical efficacy through innovative drug delivery systems. By accelerating the transition from the laboratory to production and marketing, we will be able to achieve broader clinical applications, bring real therapeutic benefits to patients, and promote the clinical popularization and application of drug delivery systems.

The field of LPs-DS harbors immense potential for innovative applications, fostering endless possibilities for exploration. It is our aspiration that this review serves as a catalyst, inspiring forthcoming research endeavors aimed at tailoring specialized LPs-DS as targeted delivery systems for varied wound types in the field of skin wound healing.
